# Cognitive Reserve as a Protective Factor for Visuospatial Ability in Healthy Aging

**DOI:** 10.3390/healthcare13233162

**Published:** 2025-12-03

**Authors:** Marika Mauti, Elena Allegretti, Raffaella I. Rumiati

**Affiliations:** 1 Department of Psychology, Sapienza University of Rome, 00185 Rome, Italy; elena.allegretti@uniroma1.it; 2 IRCCS Fondazione Santa Lucia, 00179 Rome, Italy; 3 Neuroscience Area, Scuola Internazionale Superiore di Studi Avanzati (SISSA), 34136 Trieste, Italy; 4 Department of Systems Medicine, University of Roma Tor Vergata, 00133 Rome, Italy

**Keywords:** aging, cognitive reserve, visuospatial abilities, mental rotation

## Abstract

**Background**: Cognitive Reserve (CR) is a theoretical construct developed to explain individual differences in resilience to age-related cognitive decline. Empirical evidence supports its positive role across multiple cognitive domains. However, behavioral research has primarily focused on areas either vulnerable to aging, such as memory, or relatively preserved, such as language. In contrast, the relationship between CR and task-specific performance in domains like visuospatial processing—a domain critical for everyday functioning—remains underexplored. This study investigates whether CR, as measured by the Cognitive Reserve Index Questionnaire (CRIq), predicts performance in mental rotation tasks in healthy older adults. **Methods**: Participants (age 55–85) completed two tasks: (1) a hand laterality task, requiring judgments about whether a rotated hand image (palm or back view) was left or right; and (2) a letter-congruency task, in which participants determined whether simultaneously presented rotated letters were identical or mirror-reversed. **Results**: Generalized and linear mixed-effects models revealed a protective effect of cognitive reserve, with higher CRIq scores significantly predicting greater accuracy in both tasks. Efficiency benefits (i.e., shorter reaction times) were evident mainly in the easiest conditions, suggesting that CR supports processing resources more effectively under moderate rather than maximal task demands. This pattern indicates that cognitive reserve does not uniformly enhance performance but instead modulates the allocation of cognitive resources in a context-dependent manner. **Conclusions**: To our knowledge, this is the first study to demonstrate a modulatory role of CR on visuospatial abilities in healthy older adults. These findings open new avenues for investigating how CR may differentially affect performance across a broader spectrum of cognitive functions, including attention, executive control, and spatial processing. A better understanding of these mechanisms could inform targeted cognitive interventions to strengthen resilience and promote successful aging.

## 1. Introduction

Cognitive decline is a hallmark of aging, but its onset and severity vary widely across individuals and cognitive domains [1]. While crystallized abilities, like vocabulary and verbal knowledge, are largely preserved and may even improve into the 60 s [2,3], fluid abilities—such as memory, executive functioning, processing speed, and visuospatial skills—are particularly vulnerable, and their decline can begin as early as midlife [4,5,6,7]. These changes are largely attributed to a general slowing of information processing [8], reductions in working memory capacity [9], and less efficient cognitive strategies [10], all of which can affect daily functioning. Given the absence of effective treatments, it becomes crucial to understand the mechanisms that may mitigate or buffer cognitive decline in aging and, therefore, promote healthy aging.

Visuospatial abilities—among the cognitive functions most sensitive to age-related decline [11,12,13]—are crucial for navigation, motor planning, and object manipulation. A key component is mental rotation, the ability to transform mental images of objects in space [14], a process that requires maintaining and manipulating visual representations in working memory and is therefore closely linked to visual memory [15]. In typical experiments, participants judge whether differently oriented shapes are identical or mirror images or determine body-part laterality. Reaction times increase with angular disparity across stimulus types, including 3D objects [16], alphanumeric characters [17], and body parts [18,19]. For body parts, response times match the duration of the corresponding physical movement and are slower for lateral than medial rotations—a medial–lateral effect reflecting biomechanical constraints [19].

This effect has been interpreted as evidence for motor imagery, i.e., simulating an action mentally without execution. However, growing evidence shows that participants may also use visual imagery—rotating the stimulus itself, which is not subject to the medial–lateral effect—or heuristic strategies [20,21]. Studies on brain-damaged patients demonstrate double dissociations between rotating one’s own body parts and external objects, indicating partially distinct processes [22,23]. Mental rotation is therefore multifaceted, and the underpinning mechanisms remain debated, a gap that is especially critical in the context of aging given the task’s relevance for everyday functioning. Older adults are typically slower [24,25] and less accurate, likely due to cognitive slowing, reduced efficiency of motor simulation or planning [26,27], or reliance on alternative strategies [28,29]. Age effects are strongest in hand laterality judgments, where older adults show disproportionate slowing for lateral orientations [24,25,26,27]. Additionally, while younger adults show biomechanical constraint effects mainly for palm views, older adults display greater variability and sometimes medial–lateral differences even for back views [30], suggesting a greater reliance on motor imagery over purely visuospatial strategies.

Although age-related declines in mental rotation are well established, cognitive aging is heterogeneous and does not inevitably entail deterioration. This interindividual variability underscores the importance of identifying factors that support preserved performance, among which Cognitive Reserve (CR) is particularly influential.

CR was initially proposed to explain why some individuals maintain cognitive abilities despite significant brain pathology [31]. CR represents an active model of resilience, enabling the brain to adapt dynamically to age-related and pathological changes through flexible neural reorganization and efficient cognitive strategies [31,32,33,34]. In contrast, brain reserve (BR) corresponds to a passive form of resilience, reflecting structural brain resources that provide a buffer against damage without necessarily promoting adaptive processing [31,35,36].

CR is built up throughout the lifespan via a range of life course factors such as education [37], occupational complexity [38], and engagement in intellectually stimulating activities [39,40,41]. This accumulation can be systematically assessed with the Cognitive Reserve Index questionnaire (CRIq) [42], which provides a comprehensive estimate of the reserve an individual has acquired throughout the lifespan. Research indicates that higher CR—typically measured through such proxy indicators—is associated with reduced cognitive decline and lower dementia incidence (see [43] for a review), better performance across multiple cognitive domains (e.g., verbal fluency, reasoning, executive function), slower age-related decline [44,45,46], and preserved cognitive well-being in later life [47].

Converging neuroimaging and electrophysiological evidence support the behavioral benefits of CR, showing that high CR enhances the efficiency of existing neural networks, whereas low CR involves the recruitment of additional regions under cognitive demand [48,49,50].

Most CR research has focused on domains typically affected by aging, such as memory, or on relatively preserved abilities, like language, leaving a substantial part of the cognitive spectrum unexplored. In particular, no studies have directly examined how CR may modulate mental rotation in healthy older adults, despite the well-documented age-related decline in this visuospatial domain. Given that impairments in these skills can compromise independence and quality of life in older adults, understanding whether and how CR protects against such decline is crucial. A recent study from our research group (in preparation) has already begun exploring this direction, showing that CR modulates strategy in action simulation; however, its role in visuospatial transformations remains unknown, and is the focus of the present study.

To fill this gap, in the present study, we administered two established mental rotation tasks—the Hand Laterality Task (HLT) and the Letter Congruency Task (LCT)—to a sample of healthy older adults. We quantify CR using the CRIq and test whether individuals with higher CR are more likely to adopt visual imagery strategies, reflected in greater accuracy and faster response times, when performing these visuospatial transformations.

## 2. Method

### 2.1. Participants

The initial sample consisted of 59 participants recruited through the SONA participant recruitment system. The sample size was determined based on previous studies on mental rotation abilities in older adults [26,51]. To further ensure that our analyses were adequately powered, we conducted a post hoc power analysis using the simulation method described by [52]. The results indicated that our sample size was sufficient to detect the effects of the variables of interest. The complete simulation output and a detailed explanation of this analysis are provided in the Appendix A.1. Participants were compensated € 24 for their participation in the study. Inclusion criteria required participants to be aged between 55 and 85 years, of either sex, with normal or corrected-to-normal visual acuity. Individuals were excluded if they reported a history of neurological or psychiatric disorders or substance abuse (alcohol or drugs), and if they reported a score <23 at the MOCA test. Participants provided written informed consent before starting the experiment. Ethical approval was obtained by the SISSA’s Ethic Committee (Protocol No. 24972; UOR 80923).

In the HLT, two participants were excluded due to technical errors (i.e., an insufficient number of recorded trials caused by machine error), and three were excluded due to an overall accuracy rate below 55% as in [53,54]. The final sample comprised 54 participants (mean age = 67.52, SD = 7.16; 20 males). In the LCT two participants were excluded due to an overall accuracy rate below 55%; therefore, 57 participants were included in the final analysis (mean age = 67.03, SD = 7.28; 22 males).

### 2.2. Materials

In the HLT, stimuli consisted of 24 images of human hands (left and right; palm and back views) at six different angular orientations. These images were originally developed by [19] and later modified by [55]. Each of the six rotation angles was presented 20 times, resulting in a total of 120 trials. Half of the stimuli depicted left hands and half depicted palms. All trials were presented in a fully randomized order.

In the LCT, the letters G, R, and F, in Arial, size 40, were presented in either rotated or mirror-reversed orientations. Each letter appeared in 56 trials, for a total of 168 trials, divided into three blocks—one for each letter. In each trial, two identical letters were displayed simultaneously, one to the left and one to the right of the center of the screen. While both letters were the same character, they differed in angular orientation (e.g., one at 45° and the other at 270°) and could be either congruent (rotated only) or mirror-reversed.

### 2.3. Apparatus and Procedure

Stimuli were displayed on a Dell computer screen with a resolution of 1920 × 1080 px and a refresh rate of 60 Hz. Both tasks were implemented using PsychoPy software (version 2023.2.2), and accuracy and reaction times (RTs) were recorded.

In the HLT, each trial began with a central fixation cross displayed for 200 milliseconds, followed by the hand image, which appeared at the center of the screen for either 2 or 3 s (with half of the trials at each duration), with presentation durations counterbalanced across trials for each participant. They were instructed to respond as quickly and accurately as possible by pressing the ‘C’ key with the left index finger if they believed the hand was a left hand, or the ‘N’ key with the right index finger if they believed it was a right hand.

In the LCT, each trial began with a central fixation cross presented for 200 milliseconds, followed by the letter pair, shown for one of four durations (2.0, 2.5, 3.0, or 3.5 s), with each duration occurring equally often; durations were counterbalanced across trials for each participant. Participants were instructed to press the ‘C’ key with the left index finger whether the two letters were congruent, or the ‘N’ key with the right index finger if the letters were mirror-reversed. All trials were presented in a randomized order within each block.

No specific instructions were provided regarding how to perform the mental rotation, thereby allowing participants to adopt their preferred cognitive strategy to complete the task. Two main strategies are generally adopted in mental rotation: (i) motor imagery, involving internal simulation of one’s own hand movement, and (ii) visual imagery, involving mental rotation of the visual stimulus.

The control task was a line-length judgment involving a perceptual discrimination: participants viewed two black vertical lines, one exactly twice as long as the other, making the task trivially easy. The lines were presented laterally on a white screen for one of eight randomly assigned brief intervals (16–429 ms), with each duration presented 10 times, for a total of 80 trials. In half of the trials, the longer line appeared on the right side of the screen. Participants indicated which line was longer. Considering that line-length judgment has been associated with crystallized intelligence [56], CR is not expected to influence performance in such a basic perceptual task.

### 2.4. Measures

#### 2.4.1. Cognitive Reserve Index Questionnaire

The CRIq [42] collects demographic information (e.g., age, gender, marital status) and 20 items grouped into three sections: education, working activity and leisure time activities, each yielding a specific subscore. Education records years of formal education and any additional training undertaken across the lifespan. WorkingActivity reflects the duration (in years) of professions held during adult life, classified into five occupational levels based on cognitive demands and responsibilities: unskilled manual work, skilled manual work, skilled non-manual or technical work, professional occupation, and highly intellectual occupation. Leisure time activities assess cognitively stimulating activities performed during leisure time, including intellectual, social, and physical activities.

The CRIq total score, i.e., Cognitive Reserve Index (CRI), is the average of the three subscores, with higher scores indicating greater estimated cognitive reserve. To control for age effects, scores are computed using linear regression models with age as the independent variable and raw scores as dependent variables. Residuals from these models are standardized to a scale with a mean of 100 and a standard deviation of 15. CRI is categorized into five levels: Low (<70), Medium–Low (70–84), Medium (85–114), Medium–High (115–130), and High (>130).

#### 2.4.2. Neuropsychological Tests

To evaluate participants’ cognitive profiles and to ensure that their cognitive functioning fell within the normal range, four standardized paper-and-pencil tests were administered. The Montreal Cognitive Assessment (MoCA) [57] is a brief screening tool for global cognitive functioning that assesses multiple cognitive domains, including attention, executive functions, memory, language, visuoconstructional skills, conceptual thinking, calculations, and orientation. The Colored Progressive Matrices (CPM) [58] is a non-verbal test of abstract reasoning and general intelligence, commonly used to assess fluid intelligence and problem-solving ability without the influence of language or educational background. The Trail Making Test (TMT) parts A and B [59] measures visual attention, cognitive flexibility, and processing speed. Part A requires connecting numbers in sequential order, while Part B involves alternating between numbers and letters in sequence, thereby engaging task-switching and executive control. The Digit Span (forward and backward) [60] is a subtest of the Wechsler Adult Intelligence Scale used to assess verbal working memory. The forward span measures attention and immediate memory capacity, while the backward span requires mental manipulation of information, reflecting working memory and executive function. Neuropsychological tests (MoCA, CPM, TMT, Digit Span) scores were used to ensure that our sample exhibited cognitive functioning within the normal range, thereby excluding the presence of cognitive impairment associated with aging (e.g., MCI or Alzheimer Disease). The descriptive statistics of these assessments, along with participants’ demographic information, are reported in the Appendix A.2.

### 2.5. Analysis

Data pre-processing. For the Hand Laterality task, out of 6480 trials (i.e., 54 participants × 120 trials), 132 trials (2.04%) were excluded due to machine error and 405 (6.25%) due to unrealistic reaction times, defined for each participant as exceeding 8 s [26,61] or falling more than two standard deviations above and below their mean. An additional 180 trials (2.78%) were removed because they involved stimulus rotations of 0° or 180°, which were not analyzed. The 0° orientation was excluded because it does not require any rotation, allowing for rapid identification through direct visual matching. The 180° orientation was also excluded because it involves a flip along the body’s longitudinal axis, which can often be resolved using alternative perceptual strategies rather than the incremental, motor-based mental rotation processes elicited by oblique orientations. This resulted in 5763 trials being retained for analysis. When split by Hand Orientation, we have a total of 2793 trials for the lateral condition (by participant average = 51.7 ± 5.72) and 2970 trials for the medial condition (by participant average = 55 ± 5.30). When split by Hand View, we have a total of 2920 trials for the back condition (by participant average = 54.1 ± 5.52) and 2843 trials for the palm condition (by participant average = 52.6 ± 5.61). For the Letter Congruency task, out of 9576 trials (i.e., 57 participants × 168 trials), 586 trials (6.12%) were excluded for the same reaction-time criterion, leaving 8990 trials for analysis. When split by Letter Orientation, there were 4518 trials in the congruent condition (participant mean = 79.3 ± 3.17) and 4472 trials in the mirror-reversed condition (participant mean = 78.5 ± 3.67).

Statistical analyses. Task performance was analyzed using linear and generalized linear mixed-effects models (LMMs/GLMMs; [62,63]), implemented in the lme4 R package (version 1.1-37) [64]. Separate models were run for the Hand Laterality task and the Letter Congruency task, predicting both response accuracy (binomial, 1 = correct, 0 = incorrect) and reaction time (continuous). For both tasks, predictors included the Cognitive Reserve Index (CRI, continuous, range = 92–167, z-scored), and participant sex (female vs. male). We opted for the continuous CRI score rather than the five categorical levels defined in the CRIq since group sizes were highly unbalanced in our sample (high: 35 participants; medium-high: 17 participants; medium: 2 participants). Prior to analysis, CRI scores were z-scored to improve model convergence and avoid scaling issues. For the Hand Laterality task, fixed effects also included the Hand Orientation (medial vs. lateral) and the Hand View (back vs. palm). For the Letter Congruency task, we also included the Distance in the letters’ angular rotation (i.e., 45, 90, 135, 180, 225, 270, 315) and the Orientation of the two letters (congruent vs. mirror-reversed). Participants (54 in the hand task, 59 in the letter task) and stimulus duration (2 or 3 s for the hand task; 2, 2.5, 3, or 3.5 s for the letter task) were modelled as random intercepts. In the Letter Congruency task, letter type (G, R, F) was also included as a random intercept. Models were initially specified with the maximal fixed- and random-effect structure [65], including all main effects and interactions. They were then simplified using backward selection via the step() function from the lmerTest package [66], retaining the most parsimonious model that adequately captured the data [67]. Final model outputs report fixed-effect estimates with 95% confidence intervals (as effect size proxies; [68]), *t*-values (or z-values for binomial outcomes), and *p*-values from F-tests based on the Satterthwaite approximation [69]. Pairwise comparisons were obtained using the emmeans R package [70], and their z-scores and *p*-values are directly reported in the text to establish the direction of effects in interaction terms. The original data presented in the study are openly available on the OSF data repository at https://osf.io/6jcuv (accessed on 20 November 2025).

## 3. Results

### 3.1. Control Task

Mean accuracy did not significantly differ between participants with high (96% ± 3) and low (95% ± 6) cognitive reserve, based on a median split of their continuous CRI (t(49) = 1.08, *p* = 0.29). This confirms that the two groups are comparable in perceptual discrimination ability. Thus, any interaction that might be observed between cognitive reserve and mental-rotation performance is unlikely to reflect pre-existing group differences, but rather mechanisms specifically engaged by the mental-rotation task and modulated by CR.

### 3.2. Hand Laterality Task

**Figure 1:**
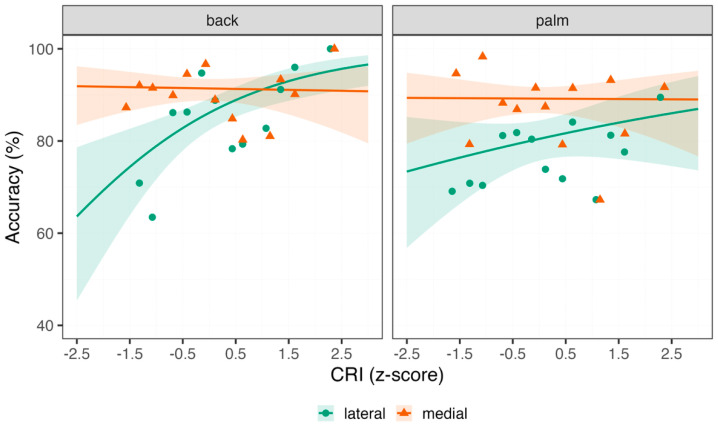
Response accuracy in the Hand Laterality task. Percentage Accuracy (y-axis) as a function of the Cognitive Reserve Index (CRI, x-axis) and the Hand Orientation (lateral = green circles; medial = orange triangles), separated by back (left panel) and right (right panel) Hand View. Raw data was binned along CRI in 14 regularly spaced bins (0.3 interval). Individual points represent the average response accuracy across trials and participants, with lines indicating the estimates of the linear models fit the data, and the shaded bands representing the 95% confidence intervals. Higher CRI is associated with better performance in the more demanding lateral orientations, especially for the back view, while medial orientations show little CRI-related change, indicating that cognitive reserve mainly benefits more effortful visuospatial/motor-imagery transformations.

Response accuracy. Performance in the Hand Laterality task increased with higher CRI, supporting the notion that cognitive reserve facilitates more efficient and flexible cognitive processing. As expected, medially oriented hands were judged more accurately than laterally oriented ones, consistent with the well-documented medial advantage, which reflects reduced visuo-spatial demands for medial orientations. Stimuli depicting the palm were judged less accurately than those showing the back of the hand, in line with the biomechanical constraint effect. Significant two-way interactions showed that the medial-lateral and back-palm accuracy gaps were largest among participants with lower CRI and gradually diminished with increasing CRI. This pattern suggests that higher cognitive reserve attenuates mental rotation costs. A significant three-way interaction further indicated that CRI moderated the orientation effect differently for back and palm views. Specifically, the reduction in the medial advantage at higher CRI was more pronounced when judging the back of the hand than the palm (Figure 1). See Table 1 for model coefficients.

**Figure 2:**
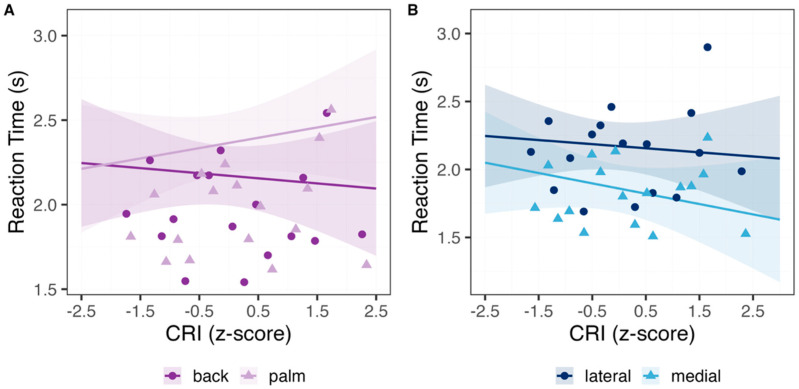
Reaction Times in the Hand Laterality task. Reaction Times in seconds (y-axis) as a function of the CR index (x-axis), shown separately for (**A**) the Hand View (back = dark purple, palm = light purple) and (**B**) the Hand Orientation (lateral = navy blue, medial = light blue). Raw data was binned along CRI in 18 regularly spaced bins (0.2 interval), ranging from −3.03 to 2.33. Individual points represent the average response accuracy across trials and participants, with lines indicating the estimates of the linear models fit the data, and the shaded bands representing the 95% confidence intervals. RTs are similar for back and palm views at low CRI, but with increasing CRI, they remain stable for back views and become slower for palm views (panel (**A**)), suggesting more deliberate processing of less canonical palm views. Higher CRI also speeds responses for medial orientations, while lateral RTs remain largely unchanged (panel (**B**)), indicating facilitated performance in the biomechanically easier medial orientations.

Reaction times. Reaction times during successful trials (N = 4818) of the Hand Laterality task were faster for medially oriented hands compared to laterally oriented hands. Post hoc comparison revealed that this pattern was more pronounced especially for female participants (z-score = −14.55, *p* < 0.001) than males (z-score = −6.41, *p* < 0.001). Moreover, responses were slower for hands shown from the palm, compared to those shown from the back view, an effect that was significant in females (z-score = −3.52, *p* = 0.002) and showed an opposite trend in males (z-score = 2.41, *p* = 0.07). The interaction between the view and the orientation of the hand was also significant, with faster responses for the back rather than palm in the lateral condition (z-score = −3.1, *p* = 0.01) and the opposite pattern in the medial orientation (z-score = 2.99, *p* = 0.01). Although CRI alone did not significantly predict reaction times, significant two-way interactions indicated that the relationship between CRI and RTs depended on stimulus configuration. As shown in Figure 2A, at low CRI values reaction times for back- and palm-view hands are similar. With higher CRI, times remain stable for the back view but increase for the palm view. Similarly, Figure 2B shows that higher CRI predicted slower responses for medially oriented hands, whereas RTs for laterally oriented hands were largely unaffected. See Table 2 for model coefficients.

### 3.3. Letter Congruency Task

**Figure 3:**
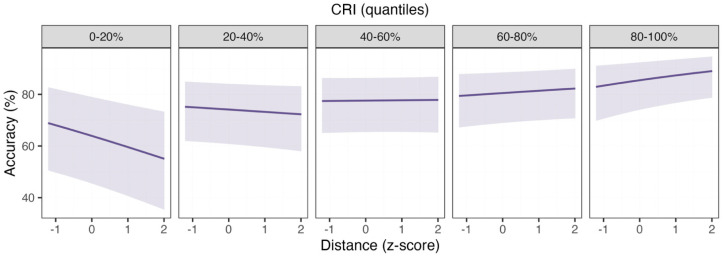
Response Accuracy in the Letter Congruency Task. Predicted Accuracy (y-axis) plotted as a function of the angular distance between the letter (x-axis) across levels of CRI organized as bins, each representing intervals of increasing CRI (i.e., −3.1/−0.79, −0.79/−0.36, −0.36/0.29, 0.29/0.65, 0.65/2.31) obtained from the underlying quantile distribution (i.e., 0%, 20%, 40%, 60%, 80%, 100%). The solid line represents the estimate of a linear mixed-effects model fit to the data and the shaded area represents the corresponding 95% confidence intervals. Accuracy declines with increasing angular distance for individuals with lower CRI, whereas this effect is reduced at higher CRI levels, suggesting that greater cognitive reserve mitigates the impact of visuospatial demands on performance.

Response accuracy. In the Letter Congruency task, response accuracy was higher for stimuli presented in the mirror-reversed than in the congruent condition and increased with higher CRI. In the mirror-reversed condition, performance improved with greater angular rotation distance between the letters, while in the congruent condition the rotation angle distance had no effect. The effect of letters’ rotation on accuracy varied as a function of CRI. For participants with lower CRI, accuracy decreased as the angular distance between the letters increased (Figure 3, first panel to the left). In contrast, for those with medium or higher CRI, accuracy was stable or even increased slightly with greater distance (Figure 3, panels on the right, see Table 3 for model outputs). The steepest improvement with distance was observed in participants with the highest CRI, suggesting that greater cognitive reserve may help maintain or enhance performance when visuo-spatial demands increase.

Reaction times. In the Letter Congruency task, responses for successful trials (N = 7015) were slower in the mirror-reversed condition than in the congruent condition. In addition, response speed increased with greater angular distance between the two letters, an effect that was particularly pronounced in the mirror-reversed condition compared to the congruent condition. No other effects, including those involving CR, were observed for this measure. The full model output is provided in Table 4.

## 4. Discussion

The present study investigated whether CR, as measured by the CRIq, modulates visuospatial transformations in healthy older adults. Despite their central role in daily functioning and higher-order processes such as spatial navigation [71], spatial reasoning [72], and motor planning [73,74], no studies have examined their relationship with CR in aging. To address this gap, we used both the HLT and the LCT and found converging evidence that higher CR is associated with more accurate performance overall and with more efficient performance, reflected in faster reaction times, though in task- and configuration-specific ways.

In the HLT, participants with higher CRI scores demonstrated greater accuracy overall. Specifically, the medial–lateral and back–palm accuracy gaps were attenuated among those with higher CRI, suggesting that CR buffers against difficulties in mentally rotating hands under demanding spatial configurations. 

The LCT results further support this interpretation: accuracy increased with higher CRI, particularly at larger angular disparities. This pattern indicates that individuals with greater reserve are better able to sustain performance as visuospatial demands intensify.

Interestingly, while CR was not a constant predictor of reaction times, its influence emerged in interaction with stimulus characteristics: consistent with existing literature [30], older adults with higher CR benefit from visual imagery in the back-of-hand condition, as shown by shorter reaction times. However, we observed a medial–lateral effect across both palm and back views, though higher CR helped mitigate this cost in the back condition. Reaction times in the LCT showed no significant association with CRI, aligning with previous evidence that cognitive reserve does not reliably influence basic or low-level processing speed [75,76]. At the same time, and in line with earlier findings [77,78], mirror-reversed trials were associated with slower responses—likely reflecting either the suppression of a prepotent “canonical” response [79] or the engagement of an additional “flip-over” sub-process [80,81]. Crucially, this cost was not moderated by CR. Consistent with previous findings, our results indicate that higher CR is associated with greater accuracy but not with faster reaction times [82]. In line with our data, previous studies have also shown that age correlates positively with RTs, with older age linked to slower responses [45,83]. In our sample, RTs were indeed positively correlated with age both in the HLT (r = 0.42, *p* = 0.001) and in the LCT (r = 0.36, *p* = 0.006), indicating that increasing age was associated with slower responses. This pattern supports the view that CR exerts its influence primarily on higher-order cognitive operations—such as flexible strategy selection and accurate mental rotation—rather than on basic perceptual speed. The latter declines robustly with age regardless of CR [3], suggesting that speed is constrained by more fundamental neurobiological changes (e.g., white matter integrity; [84]) that CR cannot easily compensate for.

Thus, CR may protect the precision and effectiveness of cognitive operations (higher accuracy) without counteracting the general slowing of responses (reaction times), which reflects structural brain aging.

Our findings contribute to prior evidence that CR supports multiple cognitive domains such as memory, attention, and language, e.g., [75,85,86,87], by showing for the first time that its protective role also extends to visuospatial transformations—a domain highly relevant to navigation, motor planning, and daily functioning, and one that shows marked decline with aging [24,25]. Evidence from other populations reinforces this broader perspective: in Parkinson’s disease, higher CR benefits executive functions [88], in healthy adults, premorbid IQ protects against age-related decline in attention, memory, and global functioning [89], and more broadly, CR has been shown to act protectively before dementia onset and compensatorily once neuropathology accumulates, e.g., [40].

In line with this broader view that targeted neural mechanisms can support higher-order cognition, recent evidence from neurofeedback interventions in schizophrenia has shown that even brief, intensive modulation of neurophysiological activity can lead to measurable improvements in cognitive performance [90], offering a proof-of-concept illustration of how neural plasticity may be harnessed to influence complex cognitive outcomes. Several caveats must be noted. The differential effects of CR on accuracy versus reaction time suggest complex underlying mechanisms, potentially involving trade-offs between motor and visual imagery strategies. Moreover, our findings cannot determine whether CR directly enhances visual imagery, promotes strategic flexibility, or both. Incorporating neuroimaging methods would allow testing whether CR moderates the relationship between brain health (e.g., gray matter volume) and cognitive performance [34]. Neurofunctional models, which posit distinct but complementary pathways through which CR shapes task performance, provide a plausible framework for interpreting these behavioral patterns. According to these accounts (e.g., [33,91]), CR supports performance through two complementary mechanisms: neural reserve, reflecting more effective use of task-relevant networks, and neural compensation, involving the flexible recruitment of additional regions when task demands increase (see [48] for a review). Although speculative, our findings are consistent with this framework. Across both the HLT and the LCT, higher CRI scores were associated with greater accuracy, particularly under more demanding spatial transformations (e.g., medial–lateral orientations and large angular disparities). This aligns with the notion that higher CR supports more efficient visuospatial processing during mental rotation, representing a behavioral expression of neural reserve. At the same time, the fact that CR reduced specific configuration-related costs (e.g., the back–palm gap in the HLT) but did not diminish age-related slowing suggests that compensatory mechanisms may selectively support higher-order visuospatial operations, whereas basic processing speed remains constrained by structural aging. Taken together, these patterns indicate that the dual-mechanism account of CR provides a coherent neurofunctional interpretation of the task- and configuration-specific benefits observed in the present study and future neuroimaging studies are needed to determine how these mechanisms unfold during visuospatial transformations. Another open question concerns the relative contribution of education, occupational complexity, and leisure activities—the CRIq subdomains—since prior evidence suggests they may differentially shape cognitive outcomes (e.g., [86,92]).

In addition, our sample was largely composed of individuals from a medium-to-high socioeconomic background, which likely implies relatively high levels of CR; future research should aim to include individuals with lower CR to better capture the full variability across the population. Moreover, the cross-sectional design of the present study prevents conclusions about causal relationships or changes over time. A longitudinal approach would be valuable to track within-person cognitive trajectories, particularly in the visuospatial domain, and to assess whether these are shaped by CR. Recent evidence [93] shows that cognitive trajectories, evaluated using the Mini-Mental State Examination (MMSE), are influenced by CR: despite the expected progression of brain deterioration, individuals with higher CR may not experience accelerated decline but rather exhibit a more gradual trajectory, maintaining an adequate level of cognitive functioning for a longer period.

Future research should also examine whether the visuospatial benefits of higher CR are linked to mental health. Emerging evidence shows that greater CR is associated with fewer symptoms of depression and anxiety, higher psychological well-being, and greater resilience to stressors [94,95,96,97]. These factors may interact with performance on demanding visuospatial tasks. Moreover, in our study, CR was conceptualized as a multidimensional construct shaped by educational attainment, occupational complexity, and engagement in enriching leisure activities, as assessed by the CRIq. Within this framework, our results on visuospatial abilities align with the broader literature demonstrating the protective influence of CR on cognitive outcomes in later life and support the view that CR is accumulated progressively across the lifespan. Building on this perspective, it becomes essential to design interventions that foster continuous engagement in cognitively stimulating experiences. Such initiatives may include guidance programs that highlight the value of lifelong learning—regardless of one’s occupational field—as well as orientation activities for students to support the selection of educational pathways that best match their interests and strengths. Promoting these forms of sustained cognitive enrichment may help strengthen cognitive reserve and enhance resilience to age-related cognitive decline, ultimately supporting cognitive functioning in older adulthood.

## 5. Conclusions

Our study provides novel evidence that cognitive reserve moderates performance in mental rotation tasks among healthy older adults. Higher CRI scores predicted greater accuracy and reduced costs associated with biomechanical and spatial complexity, extending the protective role of reserve into the visuospatial domain. Evidence from our laboratory (in preparation) further supports this role: in a complementary study, we demonstrated that high-CR older adults were more likely to preserve efficient visual strategies in judging the laterality of body parts, whereas those with lower CR predominantly relied on less efficient kinaesthetic strategies. Together, these converging results suggest that CR primarily supports the precision and flexibility of cognitive strategies across related domains—visuospatial transformations and motor imagery—rather than merely compensating for generalized age-related slowing. Methodologically, the present work establishes a broad, domain-general contribution of CR to visuospatial resilience, while our other work (in preparation) provides mechanistic insights into the strategic processes underlying motor imagery. Taken together, these findings delineate a coherent picture in which CR not only protects against age-related decline but also offers promising implications for targeted rehabilitation programs tailored to individual reserve profiles.

## Figures and Tables

**Table 1:** Generalised Linear Mixed-Effects Model output for Response Accuracy (a binomial, 0–1, incorrect vs. correct responses) as predicted by the CRI (continuous, range = −3.04–2.33, z-scored), participant Sex (female vs. male), Hand Orientation (medial vs. lateral) and Hand View (back vs. palm). Participants (54) and stimulus Duration (2 and 3 s) were the random effects introduced as intercept. Predictors that were not retained during model selection are not listed in the table. Significant *p*-values are highlighted in bold.

Predictor	β	SE	CI (2.5%; 97.5%)	z-Value	Pr (>|z|)
(Intercept)	1.83	0.15	1.54; 2.11	12.5	**<0.001**
CRI	0.51	0.14	0.22; 0.79	3.53	**<0.001**
Medial	0.54	0.11	0.32; 0.76	4.75	**<0.001**
Palm	−0.41	0.1	−0.61; −0.21	−4.06	**<0.001**
CRI × Medial	−0.53	0.11	−0.75; −0.31	−4.68	**<0.001**
CRI × Palm	−0.35	0.1	−0.54; −0.15	−3.54	**<0.001**
Medial × Palm	0.16	0.15	−0.14; 0.46	1.03	0.3
CRI × Medial × Palm	0.36	0.15	0.07; 0.66	2.39	**0.02**

Notes: The final model formula in Wilkson notation, resulting from stepwise backwards selection, is: Response Accuracy ~ CRI + Hand Orientation + Hand View + CRI:Hand Orientation + CRI:Hand View + Hand Orientation:Hand View + CRI:Hand Orientation:Hand View + (1|participant).

**Table 2:** Linear Mixed-Effects Model output for Reaction Times as predicted by the Cognitive Reserve Index (continuous, range = −3.04–2.33, z-scored), participant Sex (female vs. male), Hand Orientation (medial vs. lateral) and Hand View (back vs. palm). Participants (54) and stimulus Duration (2, 2.5, 3, or 3.5 s) were the random effects introduced as intercept. Predictors that were not retained during model selection are not listed in the table. Significant *p*-values are highlighted in bold.

Predictor	β	SE	CI (2.5%; 97.5%)	*t*-Value	Pr (>|z|)
(Intercept)	2.17	0.09	1.99; 2.35	24.19	**<0.001**
CRI	−0.03	0.07	−0.17; 0.11	−0.43	0.67
Medial	−0.31	0.04	−0.38; −0.24	−8.86	**<0.001**
Male	−0.23	0.15	−0.51; 0.06	−1.53	0.13
Palm	0.19	0.04	0.12; 0.27	5.24	**<0.001**
CRI × Palm	0.09	0.02	0.05; 0.13	4.05	**<0.001**
Medial × Male	0.18	0.05	0.09; 0.27	3.85	**<0.001**
Medial × Palm	−0.19	0.04	−0.28; −0.11	−4.39	**<0.001**
Male × Palm	−0.19	0.05	−0.28; −0.1	−4.05	**<0.001**
CRI × Medial	−0.05	0.02	−0.09; −0.00	−2.03	**0.04**

Notes: The final model formula in Wilkson notation, resulting from stepwise backwards selection, is: Reaction Times ~CRI + Hand Orientation + Sex + Hand View + CRI:Hand View + Hand Orientation:Sex + Hand Orientation:Hand View + Sex:Hand View + CRI:Hand Orientation + (1|participant).

**Table 3:** Generalized Linear Mixed-Effects Model output for Response Accuracy as predicted by the Cognitive Reserve Index (continuous, range = −3.04–2.33, z-scored), participant Sex (female vs. male), the angular Distance (i.e., 45, 90, 135, 180, 225, 270, 315) and the Orientation of the two letters (congruent vs. mirror-reversed). Participants (57) and stimulus Duration (2 and 3 s) were the random effects introduced as intercept. Predictors that were not retained during model selection are not listed in the table. Significant *p*-values are highlighted in bold.

Predictor	β	SE	CI (2.5%; 97.5%)	z-Value	Pr (>|z|)
(Intercept)	1.25	0.31	0.65; 1.86	4.06	**<0.001**
Distance	0.01	0.04	−0.07; 0.1	0.24	0.81
Mirror	0.5	0.06	0.39; 0.61	9.06	**<0.001**
CRI	0.35	0.13	0.1; 0.6	2.75	**0.006**
Male	−0.03	0.2	−0.42; 0.37	−0.13	0.9
Distance × Mirror	0.13	0.06	0.02; 0.23	2.29	**0.02**
Distance × CRI	0.1	0.04	0.03; 0.17	2.85	**0.004**
Mirror × CRI	−0.07	0.05	−0.17; 0.03	−1.34	0.18
Distance × Male	0.06	0.06	−0.05; 0.17	1.09	0.28
CRI × Male	−0.2	0.19	−0.58; 0.18	−1.04	0.3
Distance × CRI × Male	−0.1	0.05	−0.21; 0.00	−1.91	0.06

Notes: The final model formula in Wilkson notation, resulting from stepwise backwards selection, is: Response Accuracy ~Rotation + Orientation + CRI + Sex + Rotation:Orientation + Rotation:CRI + Orientation:CRI + Distance:CRI:Sex + (1|duration) + (1|letter type) + (1|participant).

**Table 4:** Linear Mixed-Effects Model output for Reaction Times as predicted by the Cognitive Reserve Index (continuous, range = −3.04–2.33, z-scored), participant Sex (female vs. male), the angular Distance (i.e., 45, 90, 135, 180, 225, 270, 315), and the Orientation of the two letters (congruent vs. mirror-reversed). Participants (57) and stimulus Duration (2 and 3 s) were the random effects introduced as intercept. Predictors that were not retained during model selection are not listed in the table. Significant *p*-values are highlighted in bold.

Predictor	β	SE	CI (2.5%; 97.5%)	*t*-Value	Pr (>|z|)
(Intercept)	2.25	0.14	1.98; 2.51	16.49	**<0.001**
Distance	−0.03	0.01	−0.06; −0.00	−2.12	**0.03**
Mirror	0.28	0.02	0.24; 0.32	13.48	**<0.001**
CRI	−0.05	0.08	−0.21; 0.1	−0.69	0.49
Distance × Mirror	−0.08	0.02	−0.12; −0.04	−4.12	**<0.001**
Distance × CRI	−0.02	0.01	−0.04; 0.00	−1.68	0.09

Notes: The final model formula in Wilkson notation, resulting from stepwise backwards selection, is: Reaction Times ~Distance + Orientation + CRI + Distance:Orientation + Distance:CRI + (1|duration) + (1|letter type) + (1|participant).

**Figure A1:**
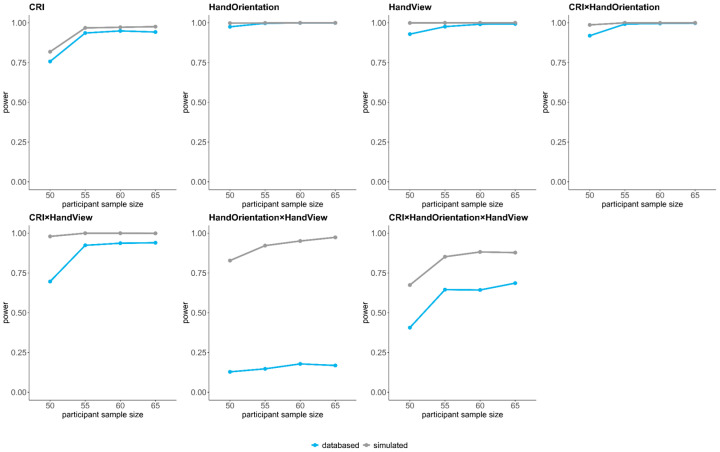
Results for power simulations using the mixedpower() function including power (y-axis) for all effects (i.e., main effects and interactions) as a function of the number of participants (x-axis). Databased (light blue) and simulated (gray) power are compared within each panel.

## Data Availability

The original data presented in the study are openly available on the OSF data repository at https://osf.io/6jcuv (accessed on 20 November 2025).

## Appendix A

### Appendix A.1. Post Hoc Simulation Based on Generalized-Linear Mixed Effect Models to Estimate Power Given Different Sample Sizes

We conducted a post hoc power analysis through simulations in the framework of generalized linear-mixed effect models following the implementation outlined by [52]. We ran separate simulations for the GLMM reported in the manuscript predicting detection accuracy in the HLT, as a function of fixed effects (CRI, Hand Orientation, Hand View) and random effects (Participants). Each simulation was run using the design matrix obtained after backward stepwise model selection, corresponding to the reduced models reported in the manuscript that retained only significant predictors, and was based on the number of observations available in our dataset (N = 5763). We used the mixedpower R package to calculate power over a range of different participants’ sample sizes (i.e., 50, 55, 60, 65). The mixedPower R package uses the data and original (G)LMM outcome to simulate new data of the same and different sample sizes used to fit the model parameters 1000 times. The power values obtained describe the proportion of how often a specific effect was found in 1000 simulated “runs” of the experiment. We computed power for all predictors by using both the effect size found in our empirical data (i.e., databased estimation, using the beta coefficients of our model) and an expected effect size for each predictor set at d = 0.2 (i.e., simulated estimation, based on adjusted effect size). We selected a d = 0.2 as it is the smallest credible effect size to detect a meaningful result. To determine Cohen’s d, we define the odds ratios of the fixed effects at 0.52 in our simulations, roughly corresponding to a d = 0.2 (see [98] for discussing the relation between the odd ratio and Cohen’s d). Following the recommendations provided by [99], we defined a t-value of 2 as our threshold of significance [62] and ran 1000 simulations. Results of the simulations for each sample size are summarized in Table A1 and Figure A1. Databased power for detection accuracy closely mirrored the strength of the observed effects, exceeding 0.93 for the main effects of CRI, Hand Orientation and Hand View and 0.65 for significant interactions. Simulated estimation also showed excellent power, suggesting that our sample size is sufficient to detect an effect size of 0.2 on detection accuracy for all the predictors. Overall, both the empirical and simulated analyses indicate that the current design provided sufficient power to detect effects of theoretical interest in both tasks.

**Table A1:** Observed and simulated power for main effects and interactions of the model structure used to predict detection accuracy in the HLT. We report power for each predictor across different sample sizes (50, 55, 60, 65). We used the function mixedpower() from the eponymous R package to run 1000 simulations.

Predictors	Type	N = 50	N = 55	N = 60	N = 65
CRI	databased	0.76	0.94	0.95	0.94
Hand Orientation	databased	0.98	1	1	1
Hand View	databased	0.93	0.98	0.99	0.99
CRI × Hand Orientation	databased	0.92	0.99	1	1
CRI × Hand View	databased	0.70	0.92	0.94	0.94
Hand Orientation x Hand View	databased	0.13	0.15	0.18	0.17
CRI × Hand Orientation × Hand View	databased	0.41	0.65	0.64	0.69
CRI	simulated	0.82	0.97	0.97	0.98
Hand Orientation	simulated	1	1	1	1
Hand View	simulated	1	1	1	1
CRI × Hand Orientation	simulated	0.99	1	1	1
CRI × Hand View	simulated	0.98	1	1	1
Hand Orientation × Hand View	simulated	0.83	0.92	0.95	0.97
CRI × Hand Orientation × Hand View	simulated	0.67	0.85	0.88	0.88

### Appendix A.2. Participant’s Descriptive Demographic and Neuropsychological Tests Score

**Table A2:** Descriptive demographic and neurocognitive characteristics of the sample. Mean values (±SD) are reported for age, cognitive reserve, assessed using the Criq and its subcomponents (education, working activity, and leisure time activities), and cognitive functioning, evaluated through the MoCA, CPM, TMT, and Digit Span. Sex distribution is reported as counts (M/F).

Variable	Mean (±SD)
Age	67.16 ± 7.34
Sex	23 M (36 F)
Criq (total)	134.72 ± 14.06
CRIq (education)	115.34 ± 12.49
CRIq (working activity)	117.33 ± 17.46
CRIq (leisure time activities)	148.24 ± 18.96
MoCA	25.55 ± 2.09
CPM	32.28 ± 3.02
TMT	56.81 ± 33.29
Digit Span Forward	5 ± 1.1
Digit Span Backward	3 ± 1.02
